# Metabolic Comorbidities in Pediatric Atopic Dermatitis: A Narrative Review

**DOI:** 10.3390/life13010002

**Published:** 2022-12-20

**Authors:** Edoardo De Simoni, Giulio Rizzetto, Elisa Molinelli, Guendalina Lucarini, Monica Mattioli-Belmonte, Irene Capodaglio, Gianna Ferretti, Tiziana Bacchetti, Annamaria Offidani, Oriana Simonetti

**Affiliations:** 1 Clinic of Dermatology, Department of Clinical and Molecular Sciences, Polytechnic University of Marche, 60126 Ancona, Italy; 2 Department of Clinical and Molecular Sciences-Histology, Polytechnic University of Marche, 60020 Ancona, Italy; 3 Hospital Cardiology and UTIC, Ospedali Riuniti di Ancona, 60126 Ancona, Italy; 4 Research Center of Health Education and Health Promotion, Department of Clinical Experimental Science and Odontostomatology-Biochemistry, 60126 Ancona, Italy; 5 Department of Life and Environmental Sciences-Biochemistry, Polytechnic University of Marche, 60126 Ancona, Italy

**Keywords:** atopic dermatitis, atopic eczema, pediatric population, comorbidities, obesity, dyslipidaemia, arterial hypertension

## Abstract

Atopic dermatitis (AD) is an itchy dermatitis with multifactorial aetiology, chronic-recurrent course, and typical distribution of lesions according to the age, affecting the 10–20% of pediatric population. Patients with AD, including children, suffer from many metabolic comorbidities, including metabolic syndrome, being overweight, obesity, dyslipidaemia, and arterial hypertension, all of which had a prevalence that was demonstrated to be higher than in healthy patients. The association between AD and metabolic comorbidities is multifactorial and involves the deregulation of immune system. In fact, hypertrophic adipose tissue produces soluble adipokines involved in inflammation and immunity, which stimulate the production of pro-inflammatory cytokines, responsible for a chronic low-grade inflammatory state and a higher predisposition to hypersensitivity reactions. Especially in pediatric population with AD, these metabolic disorders are usually underestimated and are associated with long term *sequelae* and an increased risk of a cardiovascular event, which may also occur later in adult age. Therefore, metabolic comorbidities should be carefully evaluated and early treated in children with AD, to minimize the long-term risk of cardiovascular events.

## 1. Introduction

Atopic dermatitis (AD) or atopic eczema is an itchy dermatitis with multifactorial aetiology, chronic-recurrent course, and a typical distribution of lesions by age [1]. AD is the most common inflammatory skin disease, with an overall prevalence of 10–20% in childhood and 2–8% in adulthood, although there is great variability in different countries [2,3].

In most of cases, AD has an onset before the age of five years with a slight male preponderance [4]; persistent AD beyond infancy may affect approximately 50% of patients diagnosed with this disease during childhood [5]. The onset in the first six months of life appears to be associated with severe disease.

The AD pathophysiology is complex, involving a strong genetic predisposition and an impaired epidermal barrier, associated with inflammatory hyper-responsiveness of the skin to different environmental factors. Although T-helper 2-mediated immune mechanisms are dominant, multiple immune pathways are involved in the genesis of this disorder [1].

Clinical examination is needed for the diagnosis of AD. It should consider the age-related distribution of eczematous lesions; in fact, infants often present with acute lesions typically involving the face, cheeks, and trunk but not the nappy area. In childhood (aged 2 years and older), eczema affects especially flexor surfaces; lastly, adolescents and adults usually have diffuse eczema or localized lesions typically affecting hands, eyelids, and flexures.

Topical therapy represents the frontline treatment of AD, both in acute phases and as maintenance. Indeed, some patients do not respond to the standard treatments, so it is necessary to considerer the role of microorganism such as *Candida* and *Malassetia*, *Dermatophytes* and *Staphylococcus aureus*, which role in aggravating AD is now well documented [6,7]. When the disease becomes severe, the switch to systemic therapy with immunosuppressive agents and/or biologic drugs is required [8].

Patients with AD suffer from a lot of comorbidities: apart from the allergic ones, numerous non-allergic comorbidities are described, suggesting the systemic nature of this cutaneous disease. These latter comprise infections, neuropsychiatric conditions, autoimmune disorders, hematologic malignancies, and metabolic diseases [9].

The prevalence of metabolic comorbidities (MC), including metabolic syndrome, being overweight, obesity, dyslipidaemia, and arterial hypertension, was demonstrated to be higher in patients with AD [9]. Furthermore, MC are associated with a higher risk of cardiovascular events, increased by the severity and duration of the underlying metabolic disorder. Therefore, especially in pediatric population, it is paramount to identify and treat metabolic diseases associated with AD.

This narrative review aims to provide an overview of metabolic comorbidities in pediatric patients with AD and to summarize biological evidence related to this association. 

## 2. Methods

The literature search was performed on MEDLINE (PubMed); the keywords were: “pediatric atopic dermatitis”, “pediatric atopic eczema”, “metabolic comorbidities”, “diabetes”, “obesity”, “dyslipidaemia”, “arterial hypertension”, “heart disease”, “metabolic syndrome”. Observational studies including pediatric patients and translational research were included. 

## 3. Obesity

Obesity is a condition characterized by excess of body fat and assessed in clinical practice throughout the Body Mass Index (BMI), which is the ratio between the weight and the square of height. Since BMI variations are related with both gender and age in the pediatric population, BMI percentile curves are used to diagnose pediatric overweight and obesity, which are defined as the conditions between the 85th and 95th percentile, and above the 95th percentile, respectively. The incidence of pediatric obesity is increasing in developed countries, and the prevalence is of 30% in the USA [10].

Epidemiological studies in adults patients with AD provided strong evidence of the interdependence between obesity and AD [11]. In fact, obesity predisposes patients to AD and amplifies inflammation; on the contrary, patients with AD tend to be more obese, also because of a more sedentary lifestyle [12,13,14,15,16,17,18]. 

In details, a retrospective case–control study including 1242 patients aged 1 to 21 years demonstrated a statistically significant association between obesity and AD (Odd Ratio [OR] 2.00; 95% Confidence Interval [95%CI]: 1.22–3.26; *p* = 0.006), which remained significant in multivariate models [12]. This study demonstrated that the effects of obesity were significant only when this pathological status persisted for 2.5 to 5 years (OR 2.64; 95%CI: 1.13–6.18; *p* = 0.03) and greater than 5 years (OR 3.40; 95%CI: 1.34–8.63; *p* = 0.01) but not at less than 0.5 years (OR 0.95; 95%CI: 0.27–3.29; *p* = 0.93) or 0.5 to 2.5 years (OR 1.98; 95%CI: 0.72–5.40; *p* = 0.18). As far as the impact of age, it has been also demonstrated that the effect of obesity was significant when starting at less than 2 years (OR 15.10; 95%CI: 1.51–151.21; *p* = 0.02) and 2 to 5 years (OR 2.58; 95%CI: 1.24–5.41; *p* = 0.01) but not at greater than 5 years (OR 1.32; 95%CI: 0.66–2.64; *p* = 0.43). Lastly, obesity was associated with increased odds of more severe AD (ordinal logistic regression; OR 2.23; 95%CI: 1.11–4.95) and of more frequent pediatrician’s office visits for AD. These data suggest that prolonged obesity and obesity early in childhood worsen the AD.

Another retrospective study conducted on 76,164 patients aged 6–7 years and 201,370 patients aged 13–14 years confirmed that overweight and obesity were associated with eczema symptoms but not with rhinoconjunctivitis symptoms, and moreover, showed that in both age groups, the increased risk of eczema symptoms affected only males [13]. In addition, underweight children and adolescents had a lower risk of eczema symptoms, thus strengthening the relationship between BMI and eczema.

This strong association was also recently confirmed by Augustin et al. in a large retrospective study enrolling 291,868 German patients under the age of 18 (OR 1.3; 95%CI: 1.3–1.4; *p* < 0.05), and by Huang et al. in a case–control study including 86,969 pediatric patients with AD and 116,564 pediatric controls 1.8 (OR 1.81; 95%CI: 1.70–1.191; *p* < 0.001) [13,14]; they also demonstrated a statistically significant association between AD and metabolic syndrome (OR 1.61), adding that it is very important to consider the wide spectrum of psychiatric and behavioral comorbidities affecting children with AD, which could themselves influence metabolic comorbidities [15].

Furthermore, prospective studies demonstrated a statistically significant association between AD and obesity or high-grade overweight. For example, in a study enrolling 13,275 patients with AD and aged 12–17 years, an OR of 1.2 emerged (95%CI: 1.2–1.7; *p* < 0.0001) [16]. Similarly, Agòn-Banzo et al. reported a difference in the BMI of patients with AD compared to the control group. They compared 239 children aged 0–14 years with 105 healthy controls in the same age group. The BMI of AD patients increased by 900 g in the subgroup aged less than 2 years, by 1000–1200 g in the subgroup aged 2–9 years, and by 2 kg in the subgroup aged 9–12 years. The most striking clinical and statistical differences were observed in participants aged 12–14 years: in this age group, the mean BMI was near the 50th percentile in healthy controls, the 75th percentile in the moderate AD subgroup (near the overweight threshold), and the 97th percentile in the severe AD subgroup (obesity range). These results indicate that the relationship between AD and obesity is a function of both age and AD severity [17].

The aforementioned studies suggest that the prevention of weight gain and reduction of body mass may contribute to decrease the incidence of AD [18].

Furthermore, all of this evidence suggests that the association between obesity and AD is multifactorial [19]. As far as the molecular mechanisms, some hypotheses may be formulated on the relationship between pediatric obesity and AD. In fact, pediatric obesity is associated with several metabolic alterations, increased oxidative stress, and represents a pro-inflammatory condition. 

Hypertrophic adipose tissue produces soluble factors involved in inflammation and immunity. Therefore, adipose tissue expansion might worsen AD. Molecules secreted by adipocytes comprise proinflammatory adipokines, such as leptin, resistin, and visfatin. Adipokynes produced and released by adipocytes increase T-cell survival and stimulate the production of pro-inflammatory cytokines, including tumor necrosis factor α, interferon γ, and interleukins 6, 12, 2, and CCL2: these together with the antimicrobial peptide (AMPs), play an important role in immune system modulation [19]. Increased secretion of these proinflammatory molecules leads to a chronic low-grade inflammatory state and may predispose patients to hypersensitivity reactions. On the other side, the secretion of adiponectin, which has anti-inflammatory properties, is significantly reduced both in obesity and AD [18]. 

The expression of the adiponectin receptor in myeloid or lymphoid immune cells promotes an anti-inflammatory cytokine (IL-10) cascade and inhibits the proliferation and survival of antigen-specific T [20]. In vitro, adiponectin has been shown to suppress the expression of inflammatory mediators (thymic stromal lymphopoietin, IL-8, carbonic anhydrase II, neuron-specific NEL-like protein 2, tumor necrosis factor-alpha, and human beta-defensin) which increase the expression of lipid biosynthetic enzymes (fatty acid synthase, 3-Hydroxy-3-methylglutaryl-coenzyme A reductase (HMG-CoA) reductase, and serine-palmitoyl transferase) and of differentiation factors, particularly phyllagrin [21].

It emerges that adiponectin may suppress the development of Th2 immune response and improve epidermal differentiation and barrier function. Furthermore, in obese patients, the expansion of adipose tissue can stretch the epidermis, leading to impairment of epidermal barrier function and activation of the innate immune cascade underlying autoimmunity. Indeed, the skin barrier defect may allow allergens and/or pathogens to enter the skin, promoting the development of the Th2 cell immune response and the consequent acute allergic inflammation, which may favor the pathogenesis of AD [22]. Moreover, a study by Bapat et al. showed that dysregulation of peroxisome proliferator-activated receptor-γ in CD4+ T cells underlies obesity-associated Th2 immunopathology [23].

However, recently this scenario was further complicated by the discovery of new cytokines involved in Th2 immunity, as epithelium-derived cytokines, namely, Interleukin 25 (IL-25), interleukin 33 (IL-33) and Thymic Stromal Lymphopoietin (TSLP), which have demonstrated to trigger Th2 immunity in response to external stimuli on skin [24,25]. Anyway, the relationship between these new mediators of Th2 response and adipokines has not been fully understood.

All these biochemical and metabolic alterations can contribute to modifications of the epidermal barrier of the skin, causing increased trans-epidermal water loss and skin dryness, and could be associated with elevated cutaneous and systemic levels of pro-inflammatory mediators. In fact, obesity is associated with alterations of the cellular composition and activity of inflammatory cells in the skin. 

It must also be stressed that additionally, AD predisposes to a more sedentary lifestyle, which may increase the risk of obesity and cardiovascular disease. Furthermore, the social and environmental contexts have demonstrated to play a role as well. As summarized in Table 1, the association between overweight/obesity and AD is strong in North America and Asia, but not in Europe. These data could be explained with several multifactorial components such as industrialization, urban living, higher levels of education, higher family incomes, smaller family size, and different definitions of overweight, obesity, and AD used in European studies [19].

**Figure 1:**
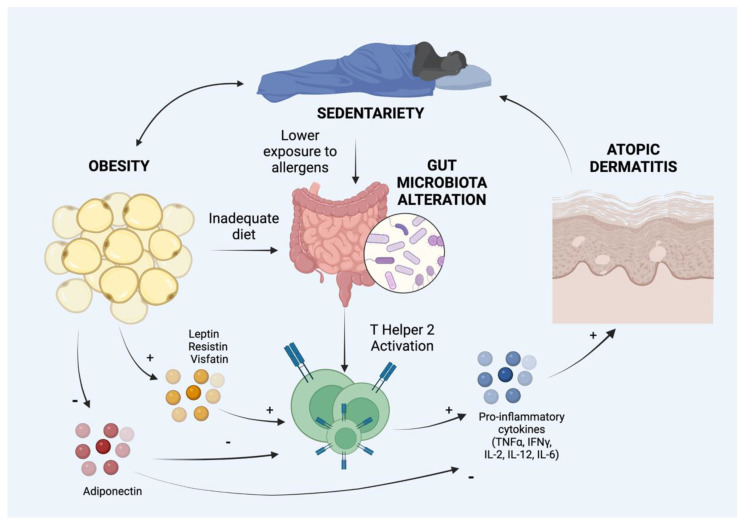
Hypothetical and demonstrated mechanisms of association between obesity and atopic dermatitis. IL: Interleukin, IFNγ: Interferon γ; TNFα: Tumor Necrosis Factor α. Created with BioRender.com, accessed on 1 September 2022.

Overweight and obesity affect the gut microbiome, which is involved in the regulation of a wide range of physiological processes (Figure 1). Furthermore, gut microbiota influences many cell-mediated immune pathways and development and the maintenance of the gut mucosa [26]. Recently it has been shown that gut microbiota plays an important role also in the development of AD both in pediatric and adult patients [27]. Therefore, we suggest that even alterations of gut microbiota could play a role in the relationship between obesity and AD [28]. In fact, the gut exposure to microbial agents in early life induces the development of a Th1-type immune population, disadvantaging the Th2 cells, which are known to predispose allergies. Furthermore, the microbiota of AD patients differs from that of non-atopic subjects, since the former are less colonized by *Enterococcous* and *Bifidobacteria* and show a higher rate of *S. aureus* and *Clostridia* in children [29]. Therefore, obese children have a more sedentary lifestyle and are consequently less exposed to allergens; furthermore, their intake of immune-modulatory nutrients (e.g., vitamin D) is reduced due to a poor and improper diet. These two factors may play a role in allergic sensitization and in the relationship between obesity and AD [30].

According to this clinical and pre-clinical evidence, there is a strong rational to evaluate the impact on the severity of AD by anti-obesity and anti-dyslipidaemia agents in obese patients with AD; however, to our knowledge, no evidence is available.

## 4. Arterial Hypertension

Arterial hypertension (AH) represents the most common cardiovascular risk factor and is a prevalent comorbidity in adult patients with AD [31].

In the pediatric population, the relationship between AH and AD is not fully understood. A retrospective study including 86,969 pediatric patients with AD and 116,564 pediatric controls failed to demonstrate a statistically significant relationship [15]. 

In contrast, a case–control study including 132 children (4–17 years) with active moderate to severe AD and 143 healthy controls demonstrated that AD was associated with AH for age, sex, and height percentiles (systolic AH: OR 2.94; 95%CI: 1.04–8.36; diastolic AH: OR 3.68; 95%CI 1.19–11.37), particularly a systolic AH in the 90th percentile or higher (OR 2.06; 95%CI: 1.09–3.90), in multivariate models that controlled for demographics, body mass index, and waist circumference percentiles, and history of using prednisone or cyclosporine [32]. In details, AD was associated with higher systolic AH in Hispanics/Latinos (general linear model; β, 0.23; 95%CI: 0.04–0.43) and Asians (β, 0.16; 95%CI: 0.03–0.30), and, as far as severity of AD is concerned, severe to very severe AD was associated with systolic AH in the 90th percentile or higher (adjusted OR 3.14; 95%CI: 1.13–8.70).

Elevated systolic blood pressure was linked to chronic inflammation and sleep impairment, both hallmarks of AD. The long-term *sequelae* of increased blood pressure are not known in children, but it is possible that cumulative increases are associated with cardiovascular disease later in life, similar to what has been observed in psoriasis. Further studies are needed to identify the triggers and mechanisms of AH in AD and to determine the long-term effects on cardiovascular health [32].

## 5. Dyslipidaemia and Atherosclerosis

Dyslipidaemia is a multifactorial disorder of the metabolism of plasma lipids and lipoproteins and represents the main risk factor of atherosclerotic disease [33].

A relationship between dyslipidaemia and AD was observed in previous studies in adult and pediatric subjects. In a prospective study comparing 239 children aged 0–14 years with 105 healthy controls age-matched, patients with severe AD had mean serum levels of all lipids higher than those observed for the AD group as a whole. However, these differences were significant only for total cholesterol. In addition, the percentage of children with elevated serum triglyceride levels was significantly higher in patients with AD, even in those with severe AD (compared with healthy controls) [17]. 

Kim et al. conducted a retrospective study of 248 children. It was found that in children with AD (n = 69, 27.8%), total cholesterol (TC), and triglyceride (TG) levels were significantly higher, but HDL-C levels were low. In addition, TC and TG were significantly associated with the SCORAD index, indicating that an abnormal blood lipid profile may contribute to the pathogenesis of AD in children [22,34].

Hyperlipidaemia can cause the release of proinflammatory cytokines and a Th2 response. In a large-scale study of adolescents (enrolled 2914), an association emerged between AD and increased LDL values emerged. Therefore, the treatment of AD in adolescents also impacts potential cardiovascular complications in adulthood [35].

A relationship between total cholesterol levels and atopy has been observed in a group of 424 patients aged 6 to 17 years [36]. Other authors reported an increased risk of allergic sensitization in adults with elevated low density lipoprotein (LDL) levels [37]; Kusunoki and colleagues observed a significant positive association between total cholesterol or LDL-C levels and allergic sensitization test results in a group of 618 11-year-old school children, but they did not observe a relationship between serum lipid concentrations and AD prevalence [38]. 

Similarly, Fessler and colleagues described a direct relationship between total cholesterol levels and atopy in a group of 424 patients aged 6 to 17 years [36]; Ouyang and colleagues reported an increased risk of allergic sensitization in adults with elevated low density lipoprotein (LDL) levels [37]; Kusunoki and colleagues observed a significant positive association between total cholesterol or LDL-C levels and allergic sensitization test results in a group of 618 11-year-old school children, but they did not observe a relationship between serum lipid concentrations and AD prevalence [37,38].

This evidence suggests a bilateral association between dyslipidaemia and AD. 

As far as the molecular mechanisms, patients with AD have been found to have increased levels of atherosclerosis markers, including fractalkine, chemokines (e.g., CCL4, CCL17, CCL28, CXCL5, CXCL10), hepatocyte growth factor (HGF), as well as increased levels of mediators of atherosclerosis, such as E-selectin or PI3/elafin, IL-16, and IL-20, which are proportional to the extension or cutaneous disease [39]. In details, fractalin is produced by endothelial cells and is a strong chemo-attractant for monocytes and lymphocytes, mediating their extravasation [40]. IL-20 is involved in hyperplasia and inhibits keratinocyte differentiation but is also expressed in atherosclerotic plaques and has been shown to promote atherosclerosis in a mouse model [41,42]; instead, IL-16, a chemo-attractant for CD4+ T-helper cells and myeloid cells, is also expressed in atherosclerotic plaques, but may have a plaque-stabilizing effect [43,44]. The role of high density lipoproteins in AD has been recently reviewed. HDL is an important modulator of the immune response. Schäfer et al. reported increased HDL-cholesterol levels in patients in comparison to controls [45]. Contrasting results have been reported by other authors [43].

All of this evidence suggests an association between dyslipidaemia and AD. Higher levels of markers of lipid peroxidation in plasma of AD pediatric patients has also been described [34]. The generation of lipid peroxidation products in plasma lipoproteins can contribute to inflammation and progression of the disease [30]. 

Recent studies have also suggested alterations of functions of high density lipoproteins in AD subjects with a significant decrease of the levels and enzymatic activity of the antioxidant and anti-inflammatory enzyme paraoxonase 1 (PON1) [46,47].

Among factors involved in the oxidative stress of plasma lipoproteins, increasing attention is devoted to the myeloperoxidase MPO enzyme, and a relationship between MPO and PON1 activity was proposed. The ratio between the serum levels of MPO and PON1 activity is described as a potential indicator of dysfunctional HDL [48,49].

A high serum MPO/PON1 ratio is described in patients affected by inflammatory diseases. Dysfunctional HDLs in AD pediatric patients are suggested also by the significant increase observed in the ratio between myeloperoxidase and PON1 (MPO/PON1) as described in our study [50]. Added authors have demonstrated alterations of HDL functions in AD with increased agonist induced eosinophil effector responses when compared to control-HDL [51,52].

## 6. Conclusions

Pediatric patients with AD are at a higher risk to develop metabolic comorbidities, such as obesity, arterial hypertension, and dyslipidaemia. These disorders are usually underestimated, and are associated with long term *sequelae* and an increased risk of cardiovascular event, which may occur also later, in adult age, as demonstrated in patients with psoriasis or lichen planus [50,53,54,55].

In our opinion, we suggest screening for metabolic comorbidities, such as dyslipidaemia, obesity, and hypertension, at the time of diagnosis of AD and during control visits, in order to intercept them early on. Adequate control and treatment of metabolic comorbidities in children is essential to reduce the risk of metabolic syndrome and cardiovascular events in adult patients. In details, at least an annual complete metabolic screening is required including biochemical examinations, adequate blood pressure measurement, and weight longitudinal monitoring.

Since the severity of AD also correlates with an increased risk of sedentary lifestyle and thus obesity, appropriate therapy must be administered, considering that the phenomenon of corticophobia often makes the disease poorly controlled in children [19,56]. The physician should periodically monitor the progression of AD in children and propose the best therapeutic solution to reduce clinical manifestations, including systemic therapy with dupilumab when necessary in severe cases [57]. Considering that the molecular mechanisms observed in development and maintenance of AD are associated with the imbalance in the levels of pro-oxidants and antioxidants, it is strongly recommended to reduce patient’s oxidative stress. For this purpose, lifestyle modifications, including dietary, physical, and psychological changes, may be effective. Thus, personalized diet modifications that emphasize fruit and vegetables intake could provide an appropriate amount of daily vitamins and bioactive molecules.

## Figures and Tables

**Table 1:** Association between AD and obesity in the pediatric population.

Author and Year	n. of pts (Age)	OR	Comments
**RETROSPECTIVE STUDIES**			
Silverberg 2011 [12]	1242 (1–21 y)	2.0 (95%CI: 1.2–3.2) to 0.8 (95%CI: 0.5–1.1) (*p* < 0.05)	Prolonged obesity increases AD Obesity early in childhood increases AD Obesity was associated with increased odds of more severe AD Obesity was associated with increased odds of more frequent pediatrician’s office visits for AD
Mitchell 2012 [13]	76,164 (age 6–7 y); 201,370 (13–14 y)	1.2 (95%CI: 1.1–1.4) to 1.4 (95%CI: 1.2–1.6) (*p* > 0.0.5)	Overweight and obesity was only associated with symptoms of eczema Increased risk of symptoms of eczema was only in males Underweight (children or adolescents) were at lower risk of symptoms of eczema
Augustin 2015 [14]	291,868 (<18 y)	1.3 (95%CI: 1.3–1.4) (*p* < 0.05)	Limited to German population
Huang 2021 [15]	86,969 (<18 y)	1.8 (95%CI: 1.70–1.191) (*p* < 0.001)	Limited to US population Median age: 4 y
**PROSPECTIVE STUDIES**			
Silverberg 2016 [16]	13,275 (12–17 y)	1.2 (95%CI: 1.2–1.7) (*p* < 0.0001)	Limited to US population
Agòn-Banzo 2019 [17]	239 (<14 y)	NA	Pediatric population from Spain BMI values were significantly higher in AD patients aged 0–2 years and 12–14 years.

Keys: AD: atopic dermatitis; BMI: Body Mass Index, CI: confidence interval; n: Number, NA: Not Available, OD: Odds Ratio; pts: patients; y: year.

## Data Availability

Not applicable.
